# Health in Persons Deprived of Their Liberty in South America: A Painful Reflection of Our Public Health

**DOI:** 10.5334/aogh.4171

**Published:** 2024-04-08

**Authors:** Franco Ernesto León-Jiménez

**Affiliations:** 1 Cesar Vallejo University, Trujillo, Peru; 2 Internal Medicine, Friendship Hospital Peru Korea Santa Rosa II-2, Piura, Peru

**Keywords:** prisoners, human rights protection, public health, correctional health, correctional center, tuberculosis, illegal substances

## Abstract

**Objectives:**

To describe sociodemographic characteristics and health-related data in persons deprived of liberty (PDL) from South America in the last five years.

**Methods:**

Documentary descriptive study.

**Results:**

There are 1.5 million PDL in Latin America and the Caribbean; the average overcrowding is 64%; 58% do not sleep in beds, 20% do not have access to clean water and 29% do not receive medical care. In Peru, during 2021, there were 87,245 PDL and 69 penal institutions. The national average overcrowding is 120%, the second-highest in South America. In South America, the prevalence of tuberculosis is 2.0% SD = 0.64 and the median of illegal substances prevalence is 34.6 (IQR = 7.5–41.4). In Peru, the prevalence of tuberculosis has decreased since 2016 (4.3%), 2018(3.5%), and 2021(2.5%). Among the health problems by country, there were more data on substance use: 8/10, and tuberculosis, 7/10 countries. Cardiovascular diseases had the least available data. Regarding COVID-19, during the first wave in Peru, 54% of the total PPL were infected, and by the end of the wave, 446 PDL and 46 members of the prison staff had died. In Colombia, between April and October 2020, there were 16,804 cases (80 in ICU) and 136 deaths. In Brazil, up to March 2021, 340 people had died, and there were over 67,000 infections.

**Conclusions:**

Overcrowding is an unresolved problem; tuberculosis and substance use are the most frequent issues. Data are limited in quality, homogeneity and availability. Greater effort is needed from health authorities to improve health management and information systematization.

**Source:**

MesH.

## Introduction

The COVID-19 pandemic, which has ravaged humanity, has highlighted the precariousness of the global public health system and its devastating impact on the most vulnerable individuals [1]. In middle- and low-income countries like ours, this reality is even more evident [2]. A special group is that of persons deprived of their liberty (PDL). The conditions in which they live—overcrowding, comorbidities, sanitation conditions, bureaucratic processes of any public entity—contribute to a compromised state of health [1,2]. Two transcendental phrases by Nelson Mandela can serve as a summarized preface for this topic: “It is said that no one truly knows a nation until one has been inside its jails. A nation should not be judged by how it treats its highest citizens, but its lowest ones [3].”

On the other hand, one of the fundamental principles of the medical profession, and of all healthcare personnel, is to offer, regardless of their beliefs, race, or social condition, the most suitable care to each person, respecting their dignity with justice and equity [4]. In persons deprived of their liberty, these aspects are often violated. Concepts such as the doctor-patient relationship [5], shared medical decision-making [6], and patient-centered medicine [7] are not always viable in the reality of these individuals.

Being a vulnerable group with decreased decision-making capacity, multiple risk factors for illness, increasing numbers of the members of this group over the years, and a potential source of medical problems with an impact on society, and with few publications on the state of their health in Latin America and particularly in our country, PDL are an important topic for a report. The objective of the study was to describe aspects related to public health in PDL in Latin America, with emphasis on Peru, during the five last years.

## Materials and Methods

Documentary research of narrative review. We conducted a search for these document types: scientific articles (Medline, BIREME, EBSCO host, Proquest, Google Scholar, Taylor and Francis, Scielo), books, and exploration of databases of the Inter-American Development Bank (https://www.iadb.org/es) [8], the International Red Cross (https://www.icrc.org/es) [9], secondary data from the Peruvian Penitentiary Institute (https://www.gob.pe/9625-acceder-a-datos-estadisticos-del-inpe) [10], and (https://siep.inpe.gob.pe/) [11]. The following keywords were used: (DeCs): *Privados de libertad, salud pública, Derechos humanos, Población vulnerable, vulnerabilidad, Establecimiento Penitenciario, Indulto, Derechos humanos, Derechos Esenciales de la Naturaleza humana, Trata Humana, Trato Digno, Gestión Penitenciaria*, and the following MeSH terms: prisoners, inmates, human rights, public health, jails.

### Ethical aspects

The data shown is available across the different databases. The Peruvian data is available as open access on the platforms of the National Penitentiary Institute of Peru: https://www.gob.pe/9625-acceder-a-datos-estadisticos-del-inpe. A final copy of the article was delivered to the Teaching and Research Unit of the Hospital de la Amistad Peru-Korea Santa Rosa II-2, Piura-Peru.

## Development

### I. Current status of the problem

#### I.1 Global demographic data

As of late 2021, there were 11.5 million PDL worldwide (93% male), with the United States leading the list with 2 million, followed by China with 1,690,000, Brazil with 811,000, and India with 478,000. In terms of incarceration rates per 100,000 inhabitants, the distribution of the top 5 is the following: The United States (629), Rwanda (580), Turkmenistan (576), El Salvador (564), and Cuba (510). The prison population has increased by 24.3% since 2020, with over 50% being in overcrowded prison systems, and 33% remaining in prison without a sentence [12,13]. This is the scenario for public health problems at a global level.

#### I.2 Demographic data in Latin America and the Caribbean

There are 1,500,000 PDL. The incarceration rate has doubled since 2000, which means that there is a 120% increase (262 per 100,000 inhabitants), compared to 24% in the rest of the world. The elderly make up 6% of the population, and the average overcrowding rate is 64%. There is approximately 45% overcrowding per cell, with 58% not sleeping in a bed, 20% lacking access to potable water, 37% without soap, and 29% not receiving medical care [14].

On April 3, 2020, the Inter-American Development Bank (IDB) organized the III International Virtual Dialogue entitled “How to address the challenges in the prison system in the context of a public health crisis: Sharing experiences and lessons learned in Latin America and the Caribbean and the world for the management of the COVID-19 pandemic.” Members of international organizations such as the International Committee of the Red Cross (ICRC), the International Corrections and Prisons Association (ICPA), the Conference of Ministers of Justice of Ibero-American Countries (COMJIB), and the Center for Studies on Innovative Prison Systems (IPS) from 15 countries in Latin America and the Caribbean participated in the dialogue as a way to harmonize solutions to this problem [15]. However, the problems persist.

#### I.3 Peruvian demographic data

In Peru, there are currently 87,245 PDL distributed in the eight regional offices of the National Penitentiary Institute, located in 25 cities and in 69 correctional centers (CC) as of 2021. According to the data from the first National Penitentiary Census (2016), in relation to the conditions of the sanitary facilities, 30.1% of PDL claimed they were “slightly clean” and 14.6% “not clean at all”; 59.7% reported that the quality of food was poor/very poor, and 17.8% reported feeling discriminated against [16]. On the other hand, in an inspection carried out by the Peruvian Ombudsman’s Office in 2018, it was found that only 64 physicians worked in the 69 CCs for a total of 82,492 PDL, a figure similar to that of 2006. Of these, 41 worked in Lima (the capital) and the region with the highest deficit was San Martin with one physician for every nine correctional centers [17]. According to the World Health Organization, a ratio of 44.5 doctors per 10,000 inhabitants would allow adequate public health care [18]. If these figures were extrapolated to PDL in Peru, there should theoretically be at least 400 doctors in charge of this population, distributed throughout the country.

Table 1 shows the sociodemographic distribution, highlighting males being most of the population, almost half the PDL as between 35–59 years old, almost 70% with a secondary education level, almost half incarcerated in Lima, and over a third population still awaiting conviction and/or sentencing. Table 1 also shows that the most overcrowded office is South Arequipa, followed by Center Huancayo.

**Table 1:** General characteristics of the prison population in Peru, 2022.


CHARACTERISTICS	TOTAL = 89,877	

	N	%

**Gender**		

Male	85,356	94.97

Female	4,521	5.03

**Age group**		

18–34	40,500	45.06

35–59	43,985	48.94

60 and over	5,392	6.0

**Educational level**		

Without formal education	1,424	1.58

Complete primary	6,969	7.75

Incomplete primary	11,007	12.25

Complete secondary	33,550	37.33

Incomplete secondary	28,610	31.83

Complete technical education	2,877	3.20

Incomplete technical education	1,900	2.11

Complete university education	1,814	2.02

Incomplete university education	1,726	1.92

**Population by Regional offices**		

North – Chiclayo	16,972	18.88

Lima – Lima	42,046	46.78

South – Arequipa	3,800	4.23

Center – Huancayo	6,707	7.46

East – Huanuco	6,459	7.19

Southeast – Cusco	5,744	6.39

Northeast – San Martin	5,577	6.21

the Andean high plateau – Puno	2,572	2.86

**Jurisdictional status**		

Prosecuted	34,071	37.91

Sentenced	55,806	62.09

**Overpopulation**		

	**Overpopulation**	**% Overpopulation**

North – Chiclayo	10,026	144

Lima – Lima	24,439	139

South – Arequipa	2,548	204

Center – Huancayo	4,460	198

East – Huanuco	2,826	99

Southeast- Cusco	3,219	97

Northeast – San Martin	225	4

the Andean high plateau – Puno	1,116	77


Source: Correctional Centers and Regional Offices; Author: National Penitentiary Institute (INPE) – Unit of Statistics.

The national overcrowding average (by regional offices) is 120% SD = 65.8.

Table 2 shows the number of CC by department and its evolution over time.

**Table 2:** Distribution of the number of correctional facilities and its evolution over time: 2011–2021.


DEPARTMENT	2011	2012	2013	2014	2015	2016	2017	2018	2019	2020	2021

Amazonas	2	2	2	2	2	2	2	2	2	2	2

Ancash	2	2	2	2	2	2	2	2	2	2	2

Apurímac	2	2	2	2	2	2	2	2	2	2	2

Arequipa	3	3	3	3	3	3	3	3	3	3	3

Ayacucho	2	2	2	2	2	2	2	2	2	2	2

Cajamarca	4	4	4	4	4	4	4	4	4	4	4

Callao	1	1	1	1	1	2	2	2	2	2	2

Cusco	4	4	4	4	4	4	4	4	4	4	4

Huancavelica	1	1	1	1	1	1	1	1	1	1	1

Huánuco	2	2	2	1	1	1	1	1	1	1	1

Ica	1	1	1	2	2	2	2	2	2	2	2

Junín	6	6	6	6	7	7	7	7	7	7	7

La Libertad	2	2	2	2	3	3	3	3	3	2	3

Lambayeque	1	1	1	1	1	1	1	1	1	1	1

Lima	13	14	13	12	12	12	12	11	11	12	12

Loreto	2	3	3	3	3	3	3	3	3	3	3

Madre de Dios	1	1	1	1	1	1	1	1	1	1	1

Moquegua	1	1	1	1	-	1	1	1	1	1	1

Pasco	1	1	1	1	1	1	2	2	2	2	2

Piura	3	3	3	3	2	2	2	2	2	2	2

Puno	3	3	3	3	3	3	3	3	3	3	3

San Martín	3	3	4	4	4	4	4	4	4	4	4

Tacna	3	3	3	3	3	3	3	3	3	3	3

Tumbes	1	1	1	1	1	1	1	1	1	1	1

Ucayali	1	1	1	1	1	1	1	1	1	1	1

**Total**	**65**	**67**	**67**	**66**	**66**	**68**	**69**	**68**	**68**	**68**	**69**


Source: Correctional Centers and Regional Offices; Author: National Penitentiary Institute (INPE) – Unit of Statistics.

As we can see, from 2011 to 2021, only four net correctional centers have been added after accounting for closings (raising the total from 65 to 69), with only Callao (1), Ica (1), Junín (1), San Martín (1), Loreto (1), La Libertad (1), and Pasco (1) increasing the number of centers. In addition, the number of facilities has decreased in Huánuco, Lima, and Piura. Arequipa, the most overcrowded, has not increased the number of correctional centers. Table 3 shows the prison population by department.

**Table 3:** Prison population by department and year.


DEPARTAMENT	2015	2016	2017	2018	2019	2020	2021

Amazonas	759	882	979	1,049	1,111	996	1,008

Ancash	3,107	3,717	4,162	4,567	4,700	4,201	4,111

Apurímac	559	631	775	912	1,039	864	923

Arequipa	2,338	2,429	2,601	2,738	2,875	2,432	2,434

Ayacucho	2,560	2,757	2,883	2,954	2,980	2,575	2,543

Cajamarca	1,793	1,935	1,970	1,999	1,986	1,797	2,237

Callao	3,303	3,315	3,056	3,096	3,228	3,093	3,248

Cusco	2,858	3,170	3,400	3,605	3,826	3,487	3,386

Huancavelica	181	220	230	247	280	209	239

Huánuco	2,500	2,939	3,116	3,231	3,370	3,015	3,098

Ica	5,494	6,607	7,078	7,300	7,794	7,242	7,189

Junín	3,019	3,175	3,466	3,903	4,004	3,528	3,655

La Libertad	4,716	5,050	5,323	5,581	5,838	5,432	5,730

Lambayeque	3 286	3 720	3 947	4 285	4 601	4 163	3 412

Lima	27,033	26,276	26,704	28,002	29,473	26,972	26,850

Loreto	1,356	1,372	1,364	1,470	1,609	1,530	1,461

Madre de Dios	747	820	894	909	1 001	950	1,015

Moquegua	–	157	227	252	271	219	242

Pasco	191	242	415	670	714	701	745

Piura	3,171	3,607	3,737	4,047	4,213	3,807	3,903

Puno	1,641	1,904	1,977	2,272	2,408	2,208	2,297

San Martín	2,547	2 699	2 807	2 970	3 158	2 935	2 930

Tacna	1,154	1,185	1,238	1,271	1,323	1,102	1,111

Tumbes	861	929	1 041	1 139	1 178	1 107	1 121

Ucayali	2,068	2,285	2,421	2,465	2,568	2,390	2,357

**Total**	**77,242**	**82,023**	**85,811**	**90,934**	**95,548**	**86,955**	**87,245**


Source: Correctional Centers and Regional Offices; Author: National Penitentiary Institute (INPE)—Unit of Statistics.

The penitentiary facility in Chanchamayo has an overpopulation rate of 477%, the highest in Peru [15].

### II. International regulatory standards

There are many documents that ensure the integrity of PDL. There are international documents and each country has its own, as well. Below is a summary of different international regulations and the sections related to public health aspects for PDL:

**International regulations on the health of persons deprived of liberty**

**1. Principles and good practices on the protection of persons deprived of liberty in the Americas, OAS/IACHR, 2008** [19]

**Principle XII:** access to an individual bed, appropriate bedding, and conditions for nighttime rest; access to hygienic sanitary facilities and private and dignified spaces. **Principle XVII:** Overcrowding is prohibited by law.

**2. Standard minimum rules of the United Nations for the treatment of prisoners (Nelson Mandela Rules), United Nations, 2015** [20]

**R12**: Having two prisoners in one cell shall be avoided.

**R13**: All sleeping accommodations shall meet all hygiene requirements.

**R18**: Personal hygiene shall be required for prisoners and they shall be provided with water and with such toilet articles.

**R22**: Every prisoner shall be provided food of nutritional value and of wholesome quality, well prepared and served.

**R24**: Prisoners should enjoy the same standards of health care that are available in the community, and should have free access to health care. Every penal facility shall have a healthcare center to assess, promote, protect, and improve physical and mental health.

**R25**: There should be an interdisciplinary team of qualified personnel and with clinical independence and mental health knowledge.

**R26**: All prisoners should be granted access to their medical files upon request. A prisoner may appoint a third party to access his or her file.

**R27**: All prisons shall ensure prompt access to medical attention in urgent cases. Those who require specialized treatment or surgery shall be transferred to specialized institutions or to civil hospitals.

**R32**: Health care professionals shall protect the physical and mental health of the prisoners and prevent and treat diseases exclusively for clinical reasons, respecting autonomy, confidentiality, and the use of the informed consent, if necessary.

**3. UNODC, WHO, UNAIDS and OHCHR joint statement on COVID-19 in prisons and other closed settings, UNODC/WHO/UNAIDS/ OHCHR, 2020** [21]

**1**. Reduce overcrowding: release prisoners with a particular risk of COVID-19, such as older people, those with comorbidities, women, and children.

**2**. Ensure decent living and working conditions and free access to healthcare services equivalent to community standards.

**3**. Ensure that prisoners continue to receive uninterrupted treatment during their detention until their release and integration into community healthcare services.

**4**. Access to telephones/digital communications if the visitation regimen is limited.

**4. Water, sanitation, hygiene, and habitat in prisons, ICRC, 2012** [22]

**1**. Penitentiary health offices shall be independent.

**2**. Minimum space per person in the room: 5.4 m^2^.

**3**. Maximum of 100 people per water faucet.

**4**. Maximum of 25 people per toilet and 50 people per shower.

**5. Bangkok Rules, UNODC, 2012 (for women)** [23]

**Rule 5**: Permanent supply of water and free sanitary towels.

**Rule 6**: Screening for STIs, mental health assessment, and assessment of violence.

**Rule 10**: Right to be evaluated by a female doctor.

**Rule 48**: Facilities to breastfeed their children.

**Rule 50**: Possibility for the child to live with their mother.

**6. Penal Execution Code, Peru, 1991** [24]

**Article 76:** Prisoners have the right to achieve, maintain, or restore physical and mental well-being. The Penitentiary Administration will provide what is necessary for this, taking into account the national policies of the Ministry of Health.

**Article 77**: Every prison has a basic medical service provided by a healthcare professional.

**Article 78:** In penitentiary centers where specialized services are justified, there will be a team of medical specialists.

**Article 79:** Penitentiary centers have areas designated for emergencies and hospitalization, with corresponding medical equipment, and isolation areas for cases of infectious diseases, psychiatric treatment, and the care of drug addicts and alcoholics.

**Article 80**: Prisoners can request, at their expense, the services of healthcare professionals external to the penitentiary center.

**Article 82**: Inmates who need specialized medical care out of the correctional center shall request it from the Penitentiary Technical Council, who have to respond to the request in no more than three days. In places where there is not the required number of physicians, the number of professionals is completed with professionals working for the state. Only in a case where there is no possibility to establish this is it completed with the available physicians.

OAS: Organization of American States; IACHR: Inter-American Commission of Human Rights; UN: United Nations; UNODC: The United Nations Office on Drugs and Crime; WHO: World Health Organization; UNAIDS: The Joint United Nations Programme on HIV/AIDS; OHCHR: Office of the United Nations High Commissioner for Human Rights; R = Mandela Rule; ICRC: International Committee of the Red Cross; STD: Sexually transmitted disease

Regarding the OAS/IACHR document, Principle XII coincides with the Mandela Rule number 13, point 4 of the joint UNODC and WHO declaration, and the Red Cross document regarding the personal space of prisoners. There has been much written about this problem and how to define it. Its presence is a risk factor for infectious diseases. The most commonly used term is overcrowding. Below we will develop some aspects found in the search for information and mentioned in the aforementioned regulations:

#### 1. Overcrowding

**Graph 1:**
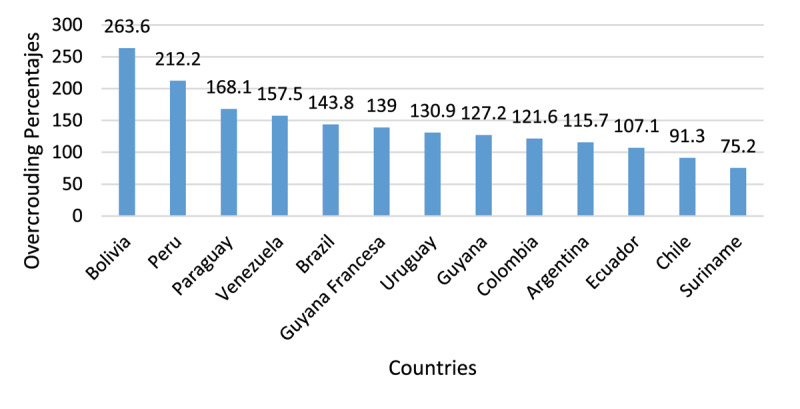
Distribution of prison overcrowding in South American countries. Source: https://www.prisonstudies.org/highest-to-lowest/occupancy-level?field_region_taxonomy_tid=24.

In the literature, the term ***hacinamiento*** (in Spanish) or overcrowding is the most commonly used by academics and politicians to refer to this issue; it is one of the most used standards for determining the severity of overpopulation. Overcrowding occurs when the prison population exceeds maximum capacity by 20% [25]. According to the Institute for Crime and Justice Policy Research at the University of London, the country with the highest overcrowding is Congo = 616.9%, followed by Haiti = 454.4% (the highest in the Americas); in South America, Bolivia tops the list with 263.6% and Peru is the second: 212.2% [12,26]. The following graph 1 shows the percentages of overcrowding by country in South America.

#### 2. Healthcare standards

According to Mandela Rule 24 and Article 76 of the Peruvian Penal Execution Code, the level of healthcare should be equal to the national standard. However, the current prison reality, overcrowding, and the assigned budget do not allow this. Nevertheless, it is known that, globally, and with greater emphasis in low- and middle-income countries, the urgent need for an approach taking into account social determinants of health and primary care is the most cost-effective measure. This could save at least 60 million lives, increase life expectancy at birth by 3.7 years, which require an injection of $200–370 billion annually. Only the improvement of global healthcare systems can impact other vulnerable groups such as PDL, and subsequently manage to make their healthcare meet the national standard [27].

#### 3. Characteristics of healthcare

As mentioned, public health in PDL in Latin America is deficient, asymmetric, and information is fragmented and heterogeneous. Below, at table 4 are some data on the prevalence of some diseases according to being infectious, chronic non-communicable, and mental health diseases in 10 Latin American countries.

**Table 4:** Frequency distribution of common health problems in persons deprived of liberty, South America.


COUNTRIES	TBC	HIV	HBP	DM	CVD	COPD/ILD	DEPRESSION	ILLEGAL SUBSTANCES	PSYCHOSIS

Argentina [40]	ND	1,7	ND	ND	ND	ND	ND	ND	ND

Bolivia [49]	2,1	ND	2,8	4,6	ND	3,11	ND	0,9	ND

Brasil [30,31,39,47,50,51,53]	2,2	1,2	24,4	3,2	ND	ND	6,9/17,6	1,3/27,9	1,1/1,4

Chile [29,36,37]	2,9	0,5	ND	ND	ND	ND	8,1	12,2	0,8

Colombia [32,33,34,35,48,52]	0,9	**11,0**	ND	ND	11,0	ND	16,7	**75,8**	3,4

Ecuador [42,43]	1,7–2,0	ND	ND	ND	ND	ND	**50,2**	41,4	**25,9**

Paraguay [41]	1,6	ND	ND	ND	ND	ND	ND	ND	ND

Perú [17,28,38]	2,5/4,3/3,5	0,4	6,9/2,0	2,8/1,7	ND	8,4	9,6	2,8	ND

Uruguay [44]	ND	ND	ND	ND	ND	ND	ND	80,0	ND

Venezuela [45,46]	ND	**4,0**	ND	ND	ND	ND	ND	56,8–57,4	ND


TBC: Tuberculosis; HBP: High blood pressure; DM: Diabetes Mellitus; CVD: Cardiovascular disease; COPD/ILD: Chronic obstructive pulmonary disease/Interstitial lung disease; ND: no data.

In regard to tuberculosis, the frequency is 2.0 (SD = 0.64). In relation to illegal substances, the summary measures are the following: median = 34.6 (IQR = 7.5–41.4). Among other striking data found, the prevalence of pulmonary tuberculosis is 81 times higher than that of the general population [14]. On the other hand, in the period between 2000–2022, in Latin America, 23 PDL died from HIV/AIDS-related causes and 1,042 from tuberculosis [28].

In Ecuador, 1 out of 5 PDL have depression/psychosis, which was found using the MINI screening tool.

The data on 4.0% frequency of HIV infection in Venezuela was based on data collected between 1998–2001. There is no additional information.

Sánchez A. et al., through a Brazilian study analyzing causes of mortality in PDL, between 2016–2017 in CC of Rio de Janeiro, found that the causes of mortality were infections (30%), heart disease (22%), and external causes (12%). Infectious causes included HIV/AIDS (43%) and tuberculosis (52%). Only 0.7% of the deceased had access to health services outside the prison. Mortality due to infections was 5 times higher, due to tuberculosis 15 times higher, and due to diabetes and heart disease 1.5 and 1.3 times higher [47].

Bolivian data are scarce, but the following stand out: According to the 2019 Prison Census, only 52% of PDL receive dinner; 18.29% of PDL do not know if there is a health area in their center, and of those who knew, only 17% knew that there was a physician and 67% knew that there was a first aid kit; only 35% received medical treatment [49].

#### 4. Peruvian data

##### 2016 Census

In regard to pharmacological treatment, the frequency with which PDL received treatment was the following: 82% of patients with HIV/AIDS, 53.1% with tuberculosis, 68.7% with diabetes, 61.6% with hypertension, 48% with chronic lung disease, 49.6% with cancer, 53.3% with depression, and 45.5% for substance use. Of a total of 28,823 episodes of illness, 78.9% were treated on an outpatient basis. The most frequent reasons for not receiving treatment were “not having money” or “lack of medication at the health center.” Moreover, at that time, the frequency of affiliation to the National Health Insurance System (SIS in spanish) was 50.5%, with a predominance in women: 71.4% vs 49.2%.

Additionally, 38.1% had an illness/disease during their stay in prison, 15.9% had difficulty seeing even with glasses, and 9.7% reported difficulty in mobilizing [16].

##### 2018 Census

In 2018, there were 3,099 cases of tuberculosis (point prevalence: 3.5%), with 73.22% being new cases, 25.01% relapses, and 1.77% recovered abandonments. Of the total cases, 6% were extrapulmonary, 46.6% of patients consumed alcohol, 64.1% used drugs, and 47.7% smoked tobacco. In 1.2% of cases, the patients already had a diagnosis of HIV before being diagnosed with tuberculosis. HIV screening was performed on 96% of patients, with a reactive result in 2.8%, “no response in the system” in 4.1%, negative in 96.7%, and “pending result evaluation” in 0.4%. Diabetes was found in 1.3% of cases through glucose testing, and 1.1% had diabetes before being diagnosed with tuberculosis [38].

##### Data from 2021

In 2021, 53,220 people received medical attention and there were 309,117 medical visits, of which 1.5% were through telemedicine. In this year there already were 33 penal facilities incorporated to the National Telehealth Network (NTN) of the Ministry of Health. By December, 83,175 (95%) people were affiliated with Comprehensive Health Insurance.

There were 2,240 cases of tuberculosis (point prevalence = 2.5%) and the region with the highest number of cases was Lima, followed by North Chiclayo. In addition, 1,305 people had mental health problems (1.4%), 1,511 had diabetes (1.7%), and 1,811 had hypertension (2.0%) [17].

As we can see, the point prevalence of tuberculosis has decreased since 2016: from 4.3 in 2016 to 3.5 in 2018 and 2.5 in 2021.

##### COVID-19 in South American prisons

According to Peruvian data from August 2020, 12,294 prisoners had been infected with COVID-19 and 212 prisoners and 15 prison officials had died as of June 2020. During the first wave, 54% of the total prison population was infected [54]. On the other hand, at the end of the first wave, according to INPE data, 446 PDL and 46 prison staff had died [55]. There are no more updated data.

In Colombia, the reality was similar. There were 16,804 cases (80 in ICU) and 136 deaths between April and October 2020 [56]. In Brazil, by the end of March 2021, 340 people had died, and there were over 67,000 cases of COVID-19 in prisons [57].

## Discussion

The prison reality in Latin America is concerning. If we accept that the characteristics of prison health should be almost equal to those of their countries of origin, there are limitations to address. Latin America is a very diverse region, with the highest inequality in the world, limitations in primary healthcare, fragmented and segmented healthcare, and therefore, the conditions of social determinants of health would partly explain why CCs do not have adequate conditions [58].

In the case of Peru, the sustained increase over time in the number of prisoners and the lack of opening of more prisons is worrying. It is known that the solution does not lie in opening more prisons but in reducing the number of prisoners. Alternatives to this, given the context of COVID-19, include release/house arrest and the stratification of each prisoner based on age, comorbidities, type of crime, terminal illness, gestational stage, and the point of completion of the sentence.

Chile (house arrest and transfer to less overcrowded prisons) and Colombia (decongestion of prisons) have already adopted measures to reduce overcrowding in prisons since 2020 [59]. Overcrowding (our country is second in South America, only behind Bolivia) is a variable associated with poor control of chronic and infectious diseases.

On the other hand, the management of prison health has many aspects that need improvement. We have not found, in the literature or national prison information, that any of the 69 national prisons have facilities for the necessary care of prisoners. The elderly and those with NCDs (6.9%: High blood pressure and 2.8%: Diabetes Mellitus) require necessary infrastructure improvements. Regarding human resources, the gap is significant, as at least 400 physicians for the 69 prisons are required. Delay times in external transfers to hospitals or health centers for surgical and acute cases are one of the limitations in the healthcare of these individuals. There is no published data on this, but it is common in hospitals for arrival times and illness times to be prolonged. This is a personal observation. However, we must mention that the continuity of treatments after hospital discharge is favored by telemedicine, present in several prisons. As of September 2021, 48/69 prisons already had this tool available [60].

Another priority area for improvement is the systematization of health data. INPE reports from different years: 2016 [16], 2018 [17], and 2021 [38] differed in some measurements due to the operational definition of diseases, such as self-report and standardized measurements, etc. This is a reality that is present in the majority of the analyzed countries. Real-time data systematization, interoperability actions, and process improvement will have an impact on measurements and, therefore, on the healthcare of these individuals. Once again, the pandemic provides an opportunity to improve these processes.

It should be noted that in the search conducted, the majority of health problems were found in Peruvian reports, except for cardiovascular diseases. Peru had the highest number of data found: 7/9, while Argentina had the highest frequency of missing data: 8/9. This could be due to differences in the budgets assigned to prison authorities or reports that could not be found during the search.

Among the health problems, substance use: 8/10, and tuberculosis: 7/10 countries, are the ones with the most information available. These two problems affect this population the most, and early diagnosis and training programs for both PDL and personnel in charge are necessary. Furthermore, given the comorbidity with HIV, early testing, case follow-up, and joint management by telemedicine with specialists (infectious disease specialists, pulmonologists, internal medicine physicians) are a priority for these patients. Long-term follow-up of pulmonary sequelae and physical therapy and rehabilitation programs are plans that should ideally be implemented as public health measures.

Cardiovascular diseases had the least available data: 1/10 countries, despite their high disease burden. This is a pending task for CC authorities.

It is noteworthy that the tuberculosis rate in Chile is the highest in South America [37]. This may be a measurement bias rather than a higher prevalence of the disease. It could imply an efficient surveillance system for this disease compared to other countries. Additionally, the prevalence of HIV in a Colombian CC was 11%, which is a highly striking number and could also be a bias since it was self-reported in 2013 in Barranquilla [34]. The prevalence of hypertension in Brazil was 24.4%, based on a self-report from 1,393 women between 2014 and 2015. This figure corresponds to that found in the general population and highlights the significant cardiovascular health problem in these women [51]. In our country, the figure is not higher than 7%, lower than the reported rate in the general population: 22% according to a meta-analysis [61]. There is likely a lack of knowledge among PDL of their hypertensive patient status, as has been reported in many studies, and this could be another self-report bias.

Regarding the use of illegal substances, it is known that Colombia is among the highest prevalence in the region. In a national survey conducted in 2019, the lifetime prevalence of illegal substance uses in the general population aged 12 to 65 years was 9.7% [62]. The data of over 70% in PDL may be due to their living conditions, the coexistence of depression, and the social determinants of health, which make these people more susceptible to this problem.

Regarding the Peruvian data on tuberculosis, a decrease in prevalence has been observed over time (2016–2021). We must take into account the different methodologies that could have been used. Active search through sputum smear microscopy and HIV screening in all positive BK patients may contribute to these figures. We should reinforce the follow-up of treated patients, implement electronic medical records for this purpose, and use telemedicine more efficiently.

Regarding COVID-19, our country has had the highest number of cases and deaths per 100,000 inhabitants in South America [63]. This is also reflected in the cited figures of this vulnerable population in our country. There are no updated comparative data on these figures in South America after the four waves of COVID-19, nor on the vaccination rate in PDL. These data are pending work. The health authorities of our country should direct efforts towards mass vaccination in PDL in order to reduce overcrowding through the measures already mentioned, and improve information management. As of October 2021, according to reports from the National Penitentiary Institute authorities, 43.2% of PDL in Peru had already received the fourth shot of the vaccine, and 100% had received at least three shots [64]. These figures are encouraging.

Among the limitations of the study, we must mention that the statistics were not processed in the same years, nor with standardized methodologies (self-report vs questionnaires vs laboratory exams). Likewise, in the vast majority of cases, the period prevalence has been one year, and in others, lifetime prevalence (e.g., mental health). Additionally, for some countries, studies with small sample sizes that do not necessarily reflect the sampling frame of that country have been chosen and could be a bias. On the other hand, there are several confounding variables that have not been addressed, such as the number of CCs, the distribution of gender, and the budgets allocated to the care in the different CCs in South America.

We believe that these results should encourage the scientific community to conduct cohort studies for the follow-up of different health indicators in PDLs, evaluate the risk factors associated with their appearance, and build better global indicators for comparison between countries in real-time.
